# Seasickness among Icelandic seamen

**DOI:** 10.1371/journal.pone.0273477

**Published:** 2022-08-26

**Authors:** Nanna Yr Arnardottir, Sigridur Sia Jonsdottir, Hannes Petersen

**Affiliations:** 1 School of Health Sciences, University of Akureyri, Akureyri, Iceland; 2 Department of Anatomy, Faculty of Medicine, University of Iceland, Reykjavik, Iceland; 3 Department of Surgery, Akureyri Hospital, Akureyri, Iceland; Swedish University of Agricultural Science, SWEDEN

## Abstract

**Introduction:**

The working environment abroad a ship is unique, with constant stimuli such as rolling of the vessel, noise, and vibration. Fishing industry is important for Icelandic economy, still the effect of seasickness-related symptoms on seamen´s health is not fully understood. Thus, the objective of this study is to explore the impact of seasickness-related symptoms, i.e., seasickness, seasickness symptoms and *mal de débarquement* on seaman´s health, and how their working environment may affect those factors.

**Methods:**

Cross-sectional data was collected from 262 seamen answering questionnaire. Majority of the seamen participated while attending a compulsory course held by the Maritime Safety and Survival Training Centre. The majority of participants were men. A chi-square test was used to detect the difference between variables.

**Results:**

The majority of seamen had experienced seasickness (87.8%) or *mal de débarquement* (85.8%). Having a history of tension headache (38.1%) and tinnitus (37.9%) was quite common. A total of 30.6% of the participants had been admitted to hospital once or more due to mishaps or accidents on land.

**Discussion:**

Seasickness and seasickness symptoms together with *mal de débarquement* are common in Icelandic seamen. Working conditions at sea are demanding and seam to affect the seamen´s health both at sea and ashore, making further research needed.

## Introduction

Motion sickness is not a disease, but manifestation of symptoms that appear when an individual finds himself in moving environment, most often due to a passive stay in a vehicle, travelling in different media of which sea is the best known [1]. Seasickness symptoms are various and include nausea, headache, cold sweats, fatigue, and vomiting [2, 3]. Seasickness is common among seamen [1, 2], as is *mal de débarquement*, *which tends to accompany seasickness* [2]. *Mal de débarquement* is a subjective perception of self-motion after exposure to passive motion, in most cases sea travel [4]. It may also be experienced after other forms of travel, for instance in a plane or motor vehicle [5]. An Icelandic study carried out on seamen in 2012 [2] revealed that 80% of Icelandic seamen experienced seasickness in their work on board ship, and a similar proportion (79%) experience*d mal de débarquement after disembarking*. *Studies from other countries have reported variable figures for the incidence of seasickness*, up to 60% of seamen [3]. The incidence of *mal de débarquement in the same group has been* 72–80% [6, 7]. Both seasickness and *mal de débarquement* vary in their impact on individuals; it is sometimes suggested that an individual may be susceptible to motion sickness, i.e., unusually sensitive to all movement that may cause symptoms of motion sickness [8–12]. All healthy individuals become motion sick, owing to sufficient motion stimuli, although up to 20% of people are believed to be more susceptible to motion sickness [9, 11]. As a rule, women report motion sickness and motion sickness susceptibility more often than men [13, 14]. Age is also a factor; motion sickness symptoms become familiar in children aged 6–12 years old and rare in people aged over 50 [3, 12, 15]. Genetics are known factor in motion sickness, as studies on twins [12] and direct genetic mapping confirm [16], the same applies to motion sickness susceptibility [17]. In addition, it has been demonstrated that patients who experience migraine are more susceptible to motion sickness [18, 19]. Motion sickness is also a synergistic disruption in many vestibular disorders, such as benign paroxysmal positional vertigo, Menière’s disease, and vestibular migraine [20].

The fisheries are a major economic sector in Iceland, in which 4,700 seamen were employed in 2019 [21]. Their working conditions at sea are demanding, with a combination of difficult weather and sea conditions creating a constantly moving environment, in which the body must adapt to. The size and design of a vessel, as well as the seamen´s workstation on board, appear to play a part in how these weather forces act on the ship, and hence on the seamen [3, 13]. The incidence of accidents at sea among seamen is high, as is incidence of accidents on land by comparison with other professions [22]. Loss of lives at sea has decreased considerably in recent years in Iceland; factors such as enhanced training of seamen at the Maritime Safety and Survival Training Centre, are likely to have contributed greatly to this development [23]. Despite their demanding working conditions, seamen are generally satisfied with their profession [24].

On fishing vessels, seamen generally work on shifts around the clock; shift patterns vary on ships of different kinds, from six-hour shifts (six hours on, six hours off) to sixteen-hour shifts (sixteen hours on, eight hours off); while seamen on small fishing boats (daytrips) work throughout the trip, up to 14 hours, but stay ashore during the night [25]. Studies from Iceland and other countries indicate that seamen often feel that they are tired [24], and their sleep is often disrupted, due to both noise and the movements of the vessel [26]. Inadequate sleep increases the likelihood of headache and can also be a cause of headache [27, 28]. Various environmental factors at sea are believed to have a negative impact on seamen´s health. These factors include vibration, noise, and heat [26]. Vibration and noise are known to induce headache [29] and have a negative impact upon sleep [30].

It is clear, that environmental and working conditions out at sea have a considerable effect upon seamen´s health. The fisheries are an important sector of the Icelandic economy, but few studies have been carried out on the interaction of health, working conditions and aspects relating to seasickness-related factors. The objective of this study is to explore the impact of seasickness and *mal de débarquement* on seamen´s health, and how their working environment may affect those factors.

## Methods

### Study design and selection of participants

The study is cross-sectional, and the sample was chosen by convenience sampling. Collection of data took place from April to June 2019. All participants attending compulsory courses held by the Maritime Safety and Survival Training Centre [31], a total of 376 seamen, were invited to participate in the study. Seamen in the Eyjafjörður region of north Iceland were also invited by email, in consultation with their employers; these totalled 180. The study was introduced via an electronic introductory letter which explained the purpose of the study and how it was to be carried out. If a seaman was interested, he/she would answer the introductory letter, and was provided with online access to the questionnaire. It was also possible to scan a QR code and answer the question on various smart devices. A total of 262 participants answered the questionnaires (47% participation; >90% participants came from the Maritime Safety and Survival Training Centre). Informed consent was elicited, and the participants were informed how the data would be used; the study was not personally identifiable, nor traceable in any way. The study was approved by the National Bioethics Committee (project no. 18–178), and notification was sent to the Data Protection Authority.

### Questionnaires

Participation entailed answering a questionnaire, which was an improved version of a questionnaire submitted to a similar group of participants in 2000 to 2002 [2]. The questionnaire comprised four parts; 1) demographic background variables, history of accidents at sea and on land, general health, and family history of diseases; 2) working conditions at sea; 3) seasickness experience (seasickness, *mal de débarquement*, and seasickness symptoms) (see S1 Appendix); 4) MSSQ short [32]. The questionnaire was in digital form (SoGoSurvey) and hosted by the University of Akureyri Research Centre.

### Statistics

Statistical processing was carried out using IBM SPSS Statistics version 25 and Microsoft Excel 2016. A Chi-square test was used to detect if variables were associated. Phi and Cramer‘s V coefficients were used to explore correlation between variables. Confidence limits of 5% (p<0.05) were applied.

Descriptive statistics were also applied to the data. In certain statistical calculations participants were divided into two age groups, <50 years (referred to as under 50) and ≥ 50 years (referred to as over 50), as age-related degeneration has an impact upon the inner ear, and hence on the experience of seasickness [33–35].

## Results

### Demographic information

A total of 262 seamen took part in the study; most of the answers were received from participants in the Maritime Safety and Survival Training Centre courses (91.6%). The average age of participants was 43.8 years (age range 17 to 84 years). The majority were males (95.8%; n = 250). All the participants were Icelandic citizens. Descriptive characteristics of seamen´s health and working conditions are shown in Table 1.

**Table 1:** Descriptive characteristics on health and work conditions.

**Age and working experience**	**n (%)**
**Age**	
≤ 25	31 (12.2)
26–35	48 (18.9)
36–45	56 (22.0)
46–55	64 (25.2)
56–65	43 (16.9)
≥ 66	12 (4.7)
**Working experience**	
< 1 year	30 (11.6)
1–5 years	27 (10.5)
> 5 years	201 (77.9)
**Working conditions**	
**Length of sea journey**	
Day tour	36 (13.8)
1–7 days	86 (33.1)
7–30 days	88 (33.8)
>30 days	50 (19.2)
**Type of vessel**	
<15 meters	30 (11.6)
15–24 meters	16 (6.2)
25–45 meters	47 (18.2)
>45 meters	165 (64.0)
**Age of vessel**	
<5 years	44 (17.0)
5–10 years	29 (11.2)
>10 years	182 (71.8)
Condition of vessel*	
Good	136 (53.3)
Average	101 (39.6)
Bad	18 (7.1)
**Workplace at vessel**	
Deck	66 (25.9)
Lower deck (closed from environmental visual cues)	79 (31.0)
Engine room	30 (11.8)
Bridge	80 (31.4)
**General health**	
**BMI categories (kg** * **m** ^**-2**^ **)**	
Normal weight^a^ (%)	51 (20.2)
Overweight^b^ (%)	116 (45.8)
Obesity^c^ (%)	86 (34.0)
**Hospital admission due to accidents on land**	
Never	181 (69.3)
1–3 times	76 (29.1)
>3 times	4 (1.5)
**Hospital admission due to accidents at sea**	
Never	215 (82.4)
1–3 times	45 (17.2)
>3 times	1 (0.4)

BMI = Body mass index.

^a^ Normal weight BMI 18.5–24.9 kg*m^-2^

^b^ Overweight BMI 25–29.9 kg*m^-2^

^c^ Obesity BMI ≥30 kg*m^-2^.

*Individual subjective vessel assessment.

### Health, age, working conditions and experience

**Fig 1:**
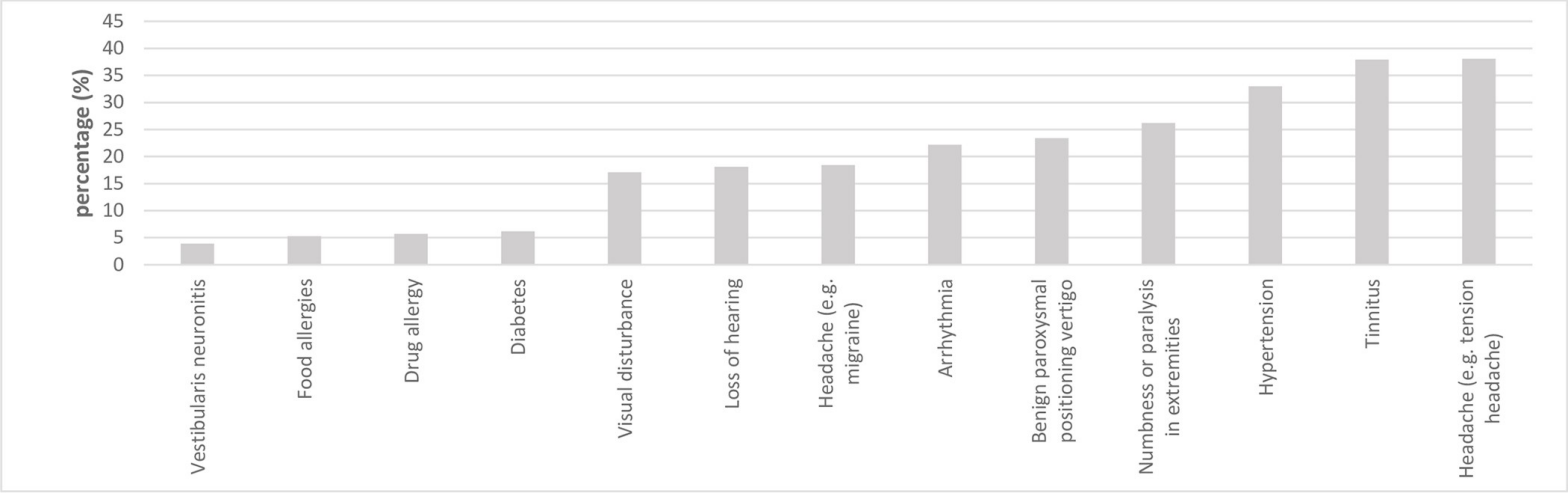
Proportion of participants that had experienced specific health conditions.

Participants were asked to specify health conditions for which they had had a diagnosis or symptoms. The findings are shown in Fig 1. According to self-reports, a total of 38.1% had a history of tension headache and 37.9% had experienced tinnitus. Hypertension had been diagnosed by a physician or symptoms of hypertension noticed among 33.0% of the seamen, and 18.4% had a history of migraine. It also turns out that 22% of seamen took over-the-counter medications for seasickness, while little use was made of prescription medications for seasickness.

### Seasickness, seasickness symptoms and *mal de débarquement*

**Fig 2:**
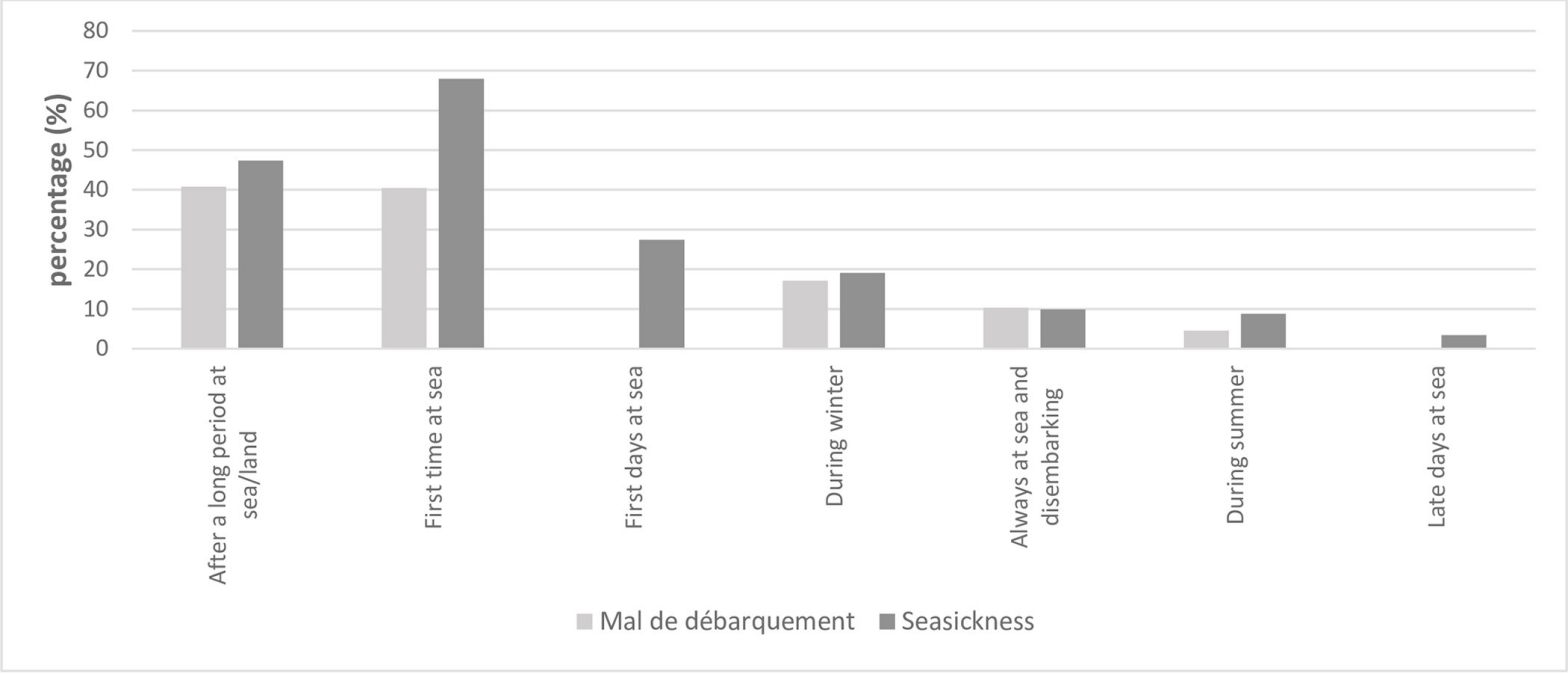
Conditions causing *mal de débarquement* and/or seasickness.

The majority of the seamen (87.8%) had experienced seasickness at some time in their lives. Of those who had experienced seasickness, most experienced it after spending a long time on land, or after their first sea journey (Fig 2). The majority (85.8%) had also experienced *mal de débarquement*; of those who had experienced *mal de débarquement*, most experienced it after a prolonged period at sea, or after their first sea journey (Fig 2). A total of 77.3% had experienced nausea, dizziness, sweats, or other symptoms relating to the movement of the ship at sea (seasickness symptoms). No difference was discerned in seasickness-related symptoms (seasickness, *mal de débarquement* or seasickness symptoms) according to where on the vessel the seaman was working, nor when the vessels were divided into upper (deck and bridge) and lower (hold and engine room) sections (p > 0.05).

### Age, length of sea journey and working experience

**Fig 3:**
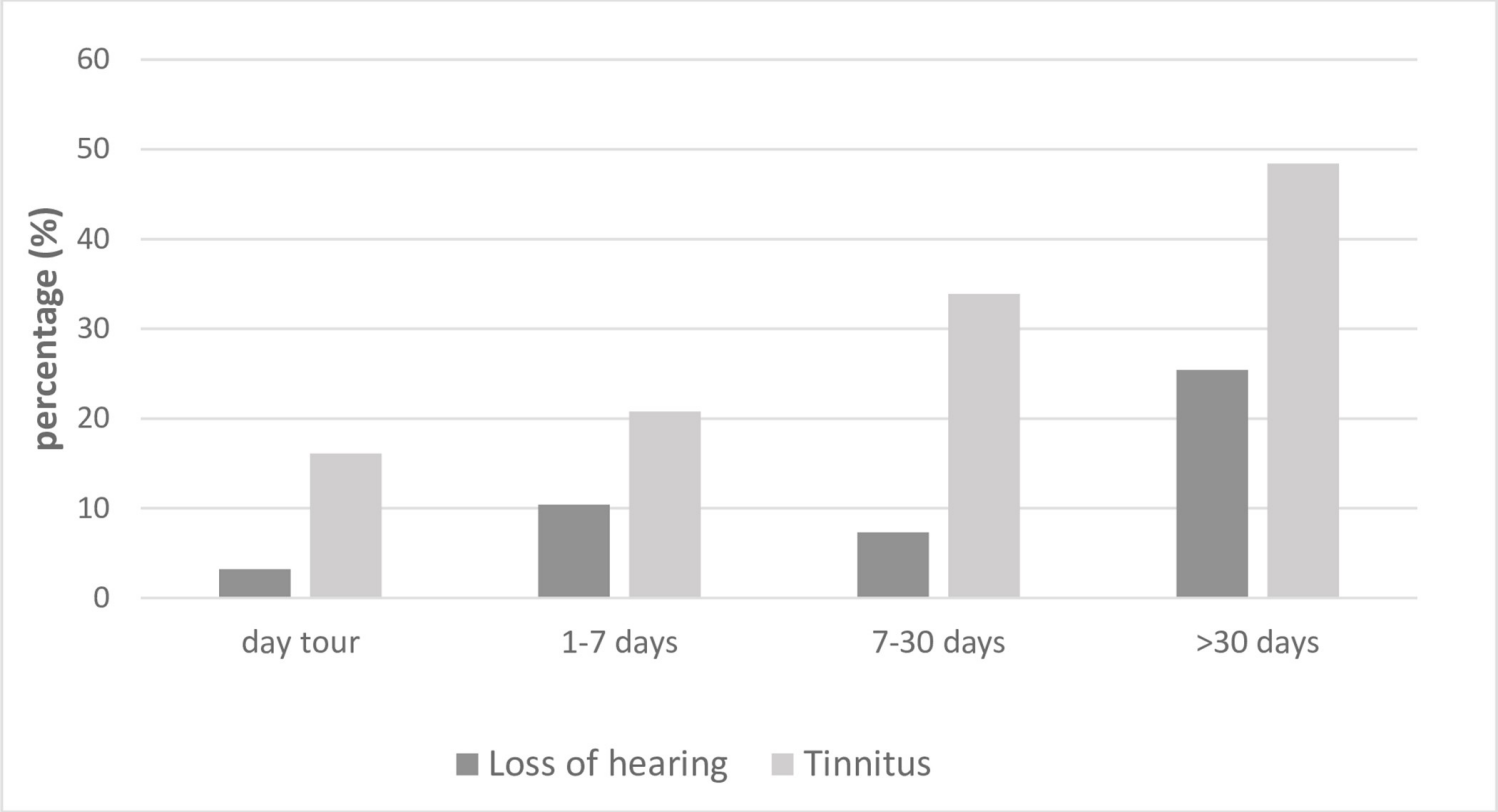
Participants who had experienced or been diagnosed with loss of hearing or tinnitus according to length of sea journey.

Seamen aged under 50 were more likely to experience seasickness symptoms at sea, compared with those aged over 50, χ^2^ (1, *n* = 254) = 7.87, p = 0.005. Significant association was found between having experienced seasickness and having experienced seasickness symptoms at sea χ^2^ (1, *n* = 262) = 11.67, p = 0.001, phi = 0.21. Seamen on smaller vessels (<45 m) were more likely to have experienced seasickness at some time in their lives χ^2^ (1, *n* = 227) = 4.26, p = 0.039 compared to those working on larger vessels. Seamen over 50 were less likely to be working on day-tour boats than those aged under 50, χ^2^ (1, *n* = 252) = 6.20, p = 0.013. Those who were out at sea for a week or less were more likely to experience seasickness symptoms than those who were out for more than a week χ^2^ (1, *n* = 259) = 6.81, p = 0.009. Pulled together, length of sea journey was not associated with seasickness, *mal de débarquement*, and experience of seasickness-symptoms. Seamen under 50 were more likely to experience tinnitus than those aged over 50 χ^2^ (1, *n* = 253) = 8.27, p = 0.004. Seamen over 50 experienced hearing loss more than those aged under 50 χ^2^ (1, *n* = 251) = 8.75, p = 0.003. Work experience at sea was associated with tinnitus χ^2^ (1, *n* = 257) = 12.72, p < 0.001, as well as hearing loss χ^2^ (3, *n* = 255) = 10.91, p = 0.012. Both tinnitus and hearing loss appear to increase in accord with length of sea journey, although no significant difference was found (p > 0.05; see Fig 3). When pulling tension headache and migraine variables, a significant association was identified between having experienced symptoms of seasickness χ^2^ (1, *n* = 257) = 7.08, p = 0.008 and *mal de débarquement* χ^2^ (1, *n* = 257) = 4.82, p = 0.028.

### Accidents at land and sea

A total of 30.6% had been admitted to hospital once or more due to accidents on land, while 17.6% had been admitted to hospital once or more due to accidents at sea (see Table 1). No difference was found between these factors and seasickness-related symptoms (all p>0.05). Seamen who had experienced tenson headache were more likely to experience accidents at sea χ^2^ (1, *n* = 256) = 7.56, p = 0.023. Scrutiny of seamen who had experienced migraine or tension headache revealed that these individuals were more likely to suffer accidents on land than those who had not experienced such symptoms χ^2^ (2, *n* = 258) = 6.09, p = 0.048. Seamen who regularly experienced tinnitus χ^2^ (2, *n* = 260) = 12.0, p = 0.002 and hearing loss χ^2^ (2, *n* = 258) = 6.75, p = 0.034, were more likely to suffer accidents at sea compared to those who did not have such symptoms.

## Discussion

The objective of the study was to explore the impact of seasickness and *mal de débarquement*, on seamen´s well-being and health, and how their working environment may affect these factors. It is distinct that seasickness and *mal de débarquement* are commonly experienced by Icelandic seamen. The incidence of accidents among seamen in Iceland is high, and headache is common. The findings of the study indicated that seasickness is as much as 8% more common that has previously been indicated for this group [2], and the same is true for *mal de débarquement*. A comparison with studies from other countries on seasickness and *mal de débarquement* indicated that the incidence is relatively high among Icelandic seamen [6, 13, 36]. Our findings indicate an increase in seasickness-related events, i.e., seasickness, *mal de débarquement* or seasickness symptoms. This may be attributable to increased knowledge of the symptoms and a broader discourse in society about seasickness; also, people today are more willing to discuss their health and well-being and working conditions than in the past. Finally, the presumption may be made that Icelandic seamen work in very demanding weather and sea conditions, even though vessels are designed to withstand environmental conditions out at sea, i.e., changing weather, temperature, and sea conditions [25]. Seamen on smaller vessels were more likely to experience seasickness symptoms; this is consistent with the study of Schutz et al., which indicates that the design of vessels is believed to have an impact on symptoms of seasickness, as seasickness is more likely on smaller vessels [13]. Smaller vessels have more agiler movements compared to larger ones, which gives rise to more energic acceleration detected by the inner ear, which in turn increase the symptoms of seasickness [37]. Our study shows that younger seamen commonly work on smaller vessels, i.e., they tolerate better the swifter moving and laborious environment experienced on board the smaller boats. Their endurance on these smaller vessels is limited as they recruit to bigger ones with increased age and time at sea. Our findings indicate that shorter tours at sea also exacerbate seasickness among seamen. These findings are not surprising, as the need for the body to adapt to frequent changes between a high-motion and a static environment is stressful and thus increases the likelihood of experiencing seasickness symptoms [38]. It transpired that seasickness declined with age, which is consistent with previous studies [9, 15]; with age the sensitivity of the inner ear declines, and this leads to decreased incidence of seasickness [33–35]. Hence it was not surprising that younger seamen, who in addition were both more likely to work on smaller vessels and taking shorter sea journeys were more likely to experience seasickness.

The incidence of accidents among the seamen who participated in the study was high, whether at sea or on land. While work-related accidents at sea are common [23], it is particularly interesting to note that 30.6% of the seamen have been admitted to hospital after accidents on land. Comparable findings have not been published before. A comparison with Iceland’s Accident Register reveals that the incidence of accidents among males in general was 11.1% in 2005–2019 [39]. Our findings also show an association between migraine/tension headache and increased likelihood of accidents, although no comparable studies have been found that indicate such an association. Migraine alone has also been associated with fatigue and drowsiness, and driving performance [28]. The incidence of migraine among seamen in the study was rather high (18.4%), but due to a lack of reports on seamen and their physical health, a comparison is difficult. Population-based surveys in Europe [40] and the USA [41] do though report similar findings. However, account being taken of the fact that the majority of the participants in our study were males, the inference may be drawn that our sample displays a higher proportion of migraine than in the European study, according to which only 8% of males experience migraine, as against 14.7% of females [40]. Comparable figures are seen in the US sample [41]. However, the incidence of tension headache in our study is comparable to the figures for men in Europe (45%) [40]. While seamen´s work involves strenuous physical activity and is often repetitive [24] it is known to give rise to increase incidence of musculoskeletal pain and associated tension headache [42].

Accidents at sea were more common among participants who had tinnitus or hearing loss. Seamen aged under 50 were more likely to experience tinnitus, while those over 50 were more likely to report hearing loss. Our findings are consistent with the known fact that increasing age, and length of service, entail increasing incidence of tinnitus. Tinnitus and hearing loss have been associated with a 25% increase in accident risk for individuals working in a noisy environment [43], and it has been demonstrated that individuals with hearing loss are more likely to sustain injuries [44].

Finally, it is important to discuss the seamen´s BMI as 80% of the participants were overweight or obese. High BMI is not a new subject, whether with respect to Icelanders in general [45] or Icelandic seamen [24]. Increased BMI does have a negative impact upon health, as it, for example, increase the likelihood of headache [46], hypertension and need for blood-pressure medication [47]. Increased BMI is also linked with poorer sleep [48].

Many of the diseases and symptoms diagnosed in the seamen or experienced by them can have a direct effect upon the experience of headache. In general, these findings lead us to consider the possible causes of this high incidence of headache. Work aboard ship is known to be demanding, with vibration, noise, poor sleep, and shift work, in addition to seasickness. It is worth considering whether the symptoms may be intensified in seamen, dwelling as they do in a constantly moving environment with constant stress-inducing noise in the environment, vibration, long shifts, and brief rest periods. It would be interesting to explore whether the motion of the vessel has an impact on seamen´s headache experience, or the working conditions as such, or a combination of the two. According to our findings, the incidence of accidents is high, and more research is required regarding its association with *mal de débarquement*. Studies are needed regarding how long after disembarking the seamen sustain accidents. In addition, it would be interesting to make a special study regarding health of female seamen, in view of their greater susceptibility to seasickness [3] and the possible impact of vibration and environmental motion on premature births [49]. The working environment abroad a ship at sea is unique. It may be viewed in a sense as a laboratory where we can research seamen´s response to stimuli found there, such as rolling of the vessel (impact of acceleration changes on the body), noise and vibration, mental challenges (stress, confined space, isolation, tedium), and finally infections in confined spaces, such as have been seen during the COVID-19 pandemic.

The strength of the study is that it was carried out using a questionnaire that has previously been used for Icelandic seamen that validate and strength our results. The cohort examined consisted of experienced seamen that had been working at sea for a relatively long time. The weakness is that the sample included few women; but the sample accurately reflect the proportion of men and women working at sea [21]. Another factor is the possibility that those who experience seasickness were more likely to participate in the study than those who do not. Participants were asked about length of service as <5 years or >5 years. It would have been interesting to be able to distinguish between seamen who had 10 to 20 years’ experience at sea, or even longer i.e., 30 to 40 years. It may also be deemed a weakness of the study that the participants themselves had to define their health conditions such as type of headache, instead of seeking permission to access medical records. Regarding seamen accidents, it is important to study the exact time of incidents on land as *mal de débarquement* might be a causative factor the first four days ashore. Understanding and attending seamen´s working environment, their health, well-being, and physical conditions is fundamental to be able to improve their working conditions and safety.

To conclude, the study indicated an increase in seasickness-related factors among Icelandic seamen compared to other studies. Icelandic seamen have a quite high admission rate to hospitals after accidents, and also report a high rate of headache, but both these factors indicate a hostile working environment where longing for safety is mandatory.

## Supporting information

S1 Appendix (DOCX)

S1 Data (SAV)
