# Supplementary material for: Seasickness among Icelandic seamen
Source: PLoS One. 2022 Aug 26;17(8):e0273477. doi: 10.1371/journal.pone.0273477 (PMC9416988; doi:10.1371/journal.pone.0273477)
Supplement: S1 Appendix — (DOCX) [file pone.0273477.s001.docx]

**Appendix 1**

| **Question** | **Answers options** |
| --- | --- |
| Gender: | Men/Women |
| Ethnicity: | Icelandic/Other |
| Age: | Please fill in |
| Hight: | Please fill in |
| Weight: | Please fill in |
| Hospital admission due to accidents on land: | Never/1-3 times/>3 times |
| Hospital admission due to accidents at sea: | Never/1-3 times/>3 times |
| Have you ever had a diagnosis or symptoms of the following: | Deafness, hearing loss (Yes/No)  Buzzing sounds in ears (Yes/No) |
| Have you ever had a diagnosis or symptoms of the following: | Meniere´s disease (Yes/No)  Vestibular neuronitis (Yes/No)  Food allergies (Yes/No)  Drug allergy (Yes/No)  Diabetes (Yes/No)  Visual disturbance (Yes/No)  Loss of hearing (Yes/No)  Headache (e.g. migraine) (Yes/No)  Arrhythmia (Yes/No)  Benign paroxysmal positioning vertigo (Yes/No)  Numbness or paralysis in extremities (Yes/No)  Hypertension (Yes/No)  Tinnitus (Yes/No)  Headache (e.g. tension headache) (Yes/No) |
|  |  |
|  |  |
|  |  |
|  |  |
|  |  |
|  |  |
|  |  |
|  |  |
|  |  |
|  |  |
|  |  |
|  |  |
|  |  |
| Are you allergic to any drugs? | Yes/No |
| *If yes:* | Please describe |
| Are you allergic to any food? | Yes/No |
| *If yes:* | Please describe |
| Are you allergic to pollen, animals or dust? | Yes/No |
| Is there a history of the following diseases in your family? | Migraine (Yes/No)  Motion sickness (Sea sickness, Car sickness, Air sickness) (Yes/No)  Cardiovascular diseases (Yes/No)  Central nervous system diseases (Yes/No)  Dizziness diseases (Yes/No) |
|  |  |
|  |  |
|  |  |
|  |  |
| Do you play video games? | Yes/No |
| *If yes:* | How often (1-2 hours/week; 4-7 hours/week; 1-2 hours/day; more than 2 hours/day) |
| Have you experienced dizziness or vertigo when playing video games? | Yes/No |
| Regarding travel outside your job: Have you ever felt nausea, dizziness, sweating or other symptoms related to movement with vehicles whether in the air, on land or at sea, when traveling? | Yes/No |
| Which vehicle has triggered motion sickness? | Car/Airplane/Train/Bus/Other |
| **Questions regarding your work at sea** |  |
| Working experience: | 1-12 months/1-2 years/2-5 years/>5 years |
| Length of sea journey: | Day tour/1-7 days/7-30 days/>30 days |
| Type of vessel: | 15-24 meters/25-45 meters/>45 meters |
| Age of vessel: | <5 years/5-10 years/>10 years |
| Condition of vessel: | Good/Average/Bad |
| Workplace at vessel: | Deck/Lower deck/Engine room/Bridge |
| Have you ever experienced nausea, dizziness, sweating or any of the symptoms associated with movement with the ship at sea? | Yes/No |
| Have you ever been seasick? | Yes/No |
| *If yes:* | First time at sea/Every time at sea/First days at sea/ Second part of sea journey/After long period at land/ During summer/During winter |
| Have you ever felt *mal de débarquement*? | Yes/No |
| *If yes:* | After first time at sea/Always after disembarking/After long period at sea/During summer/During winter |
| Regarding your work at sea, have you ever used medication to reduce sea sickness? | Yes/No |
| *If yes:* | Always before sailing/Sometimes/What medicine (describe) |
| Is there something that you do to reduce sea sickness? | Please describe |
