# Supplementary material for: Seasickness among Icelandic seamen
Source: PLoS One. 2022 Aug 26;17(8):e0273477. doi: 10.1371/journal.pone.0273477 (PMC9416988; doi:10.1371/journal.pone.0273477)
Supplement: Attachment — Submitted filename: PONE_review_renu.docx [file pone.0273477.s003.docx]

1.	The research is a simple questionnaire based cross-sectional study whose conclusions are based on responses to a 90-question questionnaire of subjects who have volunteered to participate in the project. Thus, assessment may be considered subjective depending on the state of mind and condition of individuals who have consented to provide data. To bring in objectivity, the study could have included physical examination of subjects as well as covered the assessed conclusions by validating data over a period of time.

2.	The questionnaire used has not been published along with the manuscript for the reviewer to objectively assess the interpretation of data. Also, the questionnaire has been validated not by a pilot study but by the mere improvement of an already existing questionnaire which was used in earlier research. Thus, objective validation of the questionnaire needs to be done along with a prior pilot assessment of a fixed number of subjects before undertaking the actual research. The technical soundness of the research may not be possible to comment upon since the questionnaire is not available at the present period.

3.	Although cross-sectional in nature, lack of controls in the study renders the comparative analysis of data impossible.

4.	Conclusions drawn based on the data collated reflects earlier studies in the same arena, with only a few aspects coming out as unique in the current study.

5.	Increased risk of accidents due to Mal de Debarquement within first four days after coming ashore (as declared by the authors) requires corroboration and statistical evidence which is not brought out in the current paper.

6.	Objective assessment of tension headache and tinnitus has not been done in the study and is totally based on patient description which may lead to erroneous conclusions bringing in subjectivity to the research.

7.	Having included women in the study may lead to errors in assessment, and separate criteria for assessment could have been included for women and a descriptive data analysis of impact of being at sea for women needs to be furnished. A comparative data analysis between men and women who have been at sea for the same period of time with constants of type of vessel, age profile and experience at sea may throw more light on the impact of seasickness among Icelandic ‘sea-persons’.

8.	Lines 229 to 232 are contradictory in nature. It’s a known fact that tinnitus is more common under age 50 while hearing loss is common in seamen over 50 years of age. However, the authors state the opposite. This needs to be addressed.

9.	The study concludes a higher proportion of subjects having migraine compared to a European study, however, the nature of the cited European study is obscure, viz., sample size, type of study, controls etc. Thus, this comparison is fallacious till such time both the current and European studies have been done under similar if not exact circumstances.

10.	The study purports in line 160-161 that those who were out at sea for a week or less were more likely to experience seasickness symptoms than those who were out for more than a week (p=0.009) however a contrary statement is made in line 164-167 that both tinnitus and hearing loss appear to increase in accord with length of sea journey, although no significant difference was found (p>0.05). Nor was any significant difference found between length of sea journey and seasickness-related symptoms (p>0.05). This needs deliberation.

11.	The data analysis does support the conclusion that seasickness and seasickness like symptoms along with migraine, tinnitus and tension headache are all common features in individuals who venture at sea. However, nothing new has emerged out of this questionnaire-based study except for the fact that further research needs to be done to corroborate various evidence of accidents ashore, types and sizes of vessels, duration of sea, gender differences, age variations among other factors to validate the correlation between trysts at sea with sea sickness.

12.	Statistical analysis is basic with use of SPSS ver 25. It would be prudent to bring out correlational study graphs, pie charts and a comprehensive correlational analysis to establish the soundness of the questionnaire. Further, results of Chi Square, Phi and Cramer’s V coefficient may be brought out in detail to solidify the statistical claims. Although the statistical analysis has been performed appropriately, the reviewer needs to be presented with detailed results.

13.	Authors have not made all the data underlying the findings available in the manuscript. Questionnaire is not attached and pilot survey if conducted is not described.

14.	I do not have any potential or perceived competing interests that may influence my review.

15.	No, I did not receive any assistance in preparing the review.

16.	The manuscript is presented in an intelligible fashion and written in standard English.

17.	This manuscript requires revision and re-assessment of the primary and secondary objectives prior to it being accepted and highlighted on the PLOS ONE website.
