# Supplementary material for: Seasickness among Icelandic seamen
Source: PLoS One. 2022 Aug 26;17(8):e0273477. doi: 10.1371/journal.pone.0273477 (PMC9416988; doi:10.1371/journal.pone.0273477)
Supplement: Peer review history

# Peer review: Decision Letter 0

**DOI:** 10.1371/journal.pone.0273477.r001

**Authors:** Andreas C. Bryhn

28 Apr 2022

PONE-D-21-29503

Seasickness among Icelandic seamen

PLOS ONE

Dear Dr. Arnardottir,

Thank you for submitting your manuscript to PLOS ONE. After careful consideration, we feel that it has merit but does not fully meet PLOS ONE’s publication criteria as it currently stands. Therefore, we invite you to submit a revised version of the manuscript that addresses the points raised during the review process.

Thank you for submitting a promising and interesting manuscript about seasickness to PLOS ONE. Since the three reviewers had some fundamental suggestions for improvement, I have allocated two months for you to adjust your manuscript accordingly. If you disagree with some suggestions, please respond in detail in your rebuttal document. Should you submit a revised manuscript earlier, then I will respond when you resubmit.

I also expect you to make a new search for the most recent related references, and relate your findings to those in your revised manuscript.

Please submit your revised manuscript by Jun 03 2022 11:59PM. If you will need more time than this to complete your revisions, please reply to this message or contact the journal office at plosone@plos.org. When you're ready to submit your revision, log on to https://www.editorialmanager.com/pone/ and select the 'Submissions Needing Revision' folder to locate your manuscript file.

Please include the following items when submitting your revised manuscript:

- A rebuttal letter that responds to each point raised by the academic editor and reviewer(s). You should upload this letter as a separate file labeled 'Response to Reviewers'.
- A marked-up copy of your manuscript that highlights changes made to the original version. You should upload this as a separate file labeled 'Revised Manuscript with Track Changes'.
- An unmarked version of your revised paper without tracked changes. You should upload this as a separate file labeled 'Manuscript'.

If you would like to make changes to your financial disclosure, please include your updated statement in your cover letter. Guidelines for resubmitting your figure files are available below the reviewer comments at the end of this letter.

If applicable, we recommend that you deposit your laboratory protocols in protocols.io to enhance the reproducibility of your results. Protocols.io assigns your protocol its own identifier (DOI) so that it can be cited independently in the future. For instructions see: https://journals.plos.org/plosone/s/submission-guidelines#loc-laboratory-protocols. Additionally, PLOS ONE offers an option for publishing peer-reviewed Lab Protocol articles, which describe protocols hosted on protocols.io. Read more information on sharing protocols at https://plos.org/protocols?utm_medium=editorial-email&utm_source=authorletters&utm_campaign=protocols.

We look forward to receiving your revised manuscript.

Kind regards,

Andreas C. Bryhn

Academic Editor

PLOS ONE

Journal Requirements:

When submitting your revision, we need you to address these additional requirements.

1. Please ensure that your manuscript meets PLOS ONE's style requirements, including those for file naming. The PLOS ONE style templates can be found at 

https://journals.plos.org/plosone/s/file?id=wjVg/PLOSOne_formatting_sample_main_body.pdf and 

https://journals.plos.org/plosone/s/file?id=ba62/PLOSOne_formatting_sample_title_authors_affiliations.pdf

2. Please include additional information regarding the survey or questionnaire used in the study and ensure that you have provided sufficient details that others could replicate the analyses. For instance, if you developed a questionnaire as part of this study and it is not under a copyright more restrictive than CC-BY, please include a copy, in both the original language and English, as Supporting Information.

3. In your Data Availability statement, you have not specified where the minimal data set underlying the results described in your manuscript can be found. PLOS defines a study's minimal data set as the underlying data used to reach the conclusions drawn in the manuscript and any additional data required to replicate the reported study findings in their entirety. All PLOS journals require that the minimal data set be made fully available. For more information about our data policy, please see http://journals.plos.org/plosone/s/data-availability.

Upon re-submitting your revised manuscript, please upload your study’s minimal underlying data set as either Supporting Information files or to a stable, public repository and include the relevant URLs, DOIs, or accession numbers within your revised cover letter. For a list of acceptable repositories, please see http://journals.plos.org/plosone/s/data-availability#loc-recommended-repositories. Any potentially identifying patient information must be fully anonymized.

Important: If there are ethical or legal restrictions to sharing your data publicly, please explain these restrictions in detail. Please see our guidelines for more information on what we consider unacceptable restrictions to publicly sharing data: http://journals.plos.org/plosone/s/data-availability#loc-unacceptable-data-access-restrictions. Note that it is not acceptable for the authors to be the sole named individuals responsible for ensuring data access.

We will update your Data Availability statement to reflect the information you provide in your cover letter.

4. We note that you have indicated that data from this study are available upon request. PLOS only allows data to be available upon request if there are legal or ethical restrictions on sharing data publicly. For more information on unacceptable data access restrictions, please see http://journals.plos.org/plosone/s/data-availability#loc-unacceptable-data-access-restrictions. 

In your revised cover letter, please address the following prompts:

a) If there are ethical or legal restrictions on sharing a de-identified data set, please explain them in detail (e.g., data contain potentially sensitive information, data are owned by a third-party organization, etc.) and who has imposed them (e.g., an ethics committee). Please also provide contact information for a data access committee, ethics committee, or other institutional body to which data requests may be sent.

b) If there are no restrictions, please upload the minimal anonymized data set necessary to replicate your study findings as either Supporting Information files or to a stable, public repository and provide us with the relevant URLs, DOIs, or accession numbers. For a list of acceptable repositories, please see http://journals.plos.org/plosone/s/data-availability#loc-recommended-repositories.

We will update your Data Availability statement on your behalf to reflect the information you provide.

5. We note you have included a table to which you do not refer in the text of your manuscript. Please ensure that you refer to Table 1 in your text; if accepted, production will need this reference to link the reader to the Table.

[Note: HTML markup is below. Please do not edit.]

Reviewers' comments:

Reviewer's Responses to Questions

**Comments to the Author**

1. Is the manuscript technically sound, and do the data support the conclusions?

The manuscript must describe a technically sound piece of scientific research with data that supports the conclusions. Experiments must have been conducted rigorously, with appropriate controls, replication, and sample sizes. The conclusions must be drawn appropriately based on the data presented. 

Reviewer #1: Partly

Reviewer #2: Partly

Reviewer #3: Partly

**********

2. Has the statistical analysis been performed appropriately and rigorously? 

Reviewer #1: No

Reviewer #2: No

Reviewer #3: No

**********

3. Have the authors made all data underlying the findings in their manuscript fully available?

The PLOS Data policy requires authors to make all data underlying the findings described in their manuscript fully available without restriction, with rare exception (please refer to the Data Availability Statement in the manuscript PDF file). The data should be provided as part of the manuscript or its supporting information, or deposited to a public repository. For example, in addition to summary statistics, the data points behind means, medians and variance measures should be available. If there are restrictions on publicly sharing data—e.g. participant privacy or use of data from a third party—those must be specified.

Reviewer #1: No

Reviewer #2: No

Reviewer #3: No

**********

4. Is the manuscript presented in an intelligible fashion and written in standard English?

PLOS ONE does not copyedit accepted manuscripts, so the language in submitted articles must be clear, correct, and unambiguous. Any typographical or grammatical errors should be corrected at revision, so please note any specific errors here.

Reviewer #1: Yes

Reviewer #2: Yes

Reviewer #3: Yes

**********

5. Review Comments to the Author

Please use the space provided to explain your answers to the questions above. You may also include additional comments for the author, including concerns about dual publication, research ethics, or publication ethics. (Please upload your review as an attachment if it exceeds 20,000 characters)

Reviewer #1: 1. The research is a simple questionnaire based cross-sectional study whose conclusions are based on responses to a 90-question questionnaire of subjects who have volunteered to participate in the project. Thus, assessment may be considered subjective depending on the state of mind and condition of individuals who have consented to provide data. To bring in objectivity, the study could have included physical examination of subjects as well as covered the assessed conclusions by validating data over a period of time.

2. The questionnaire used has not been published along with the manuscript for the reviewer to objectively assess the interpretation of data. Also, the questionnaire has been validated not by a pilot study but by the mere improvement of an already existing questionnaire which was used in earlier research. Thus, objective validation of the questionnaire needs to be done along with a prior pilot assessment of a fixed number of subjects before undertaking the actual research. The technical soundness of the research may not be possible to comment upon since the questionnaire is not available at the present period.

3. Although cross-sectional in nature, lack of controls in the study renders the comparative analysis of data impossible.

4. Conclusions drawn based on the data collated reflects earlier studies in the same arena, with only a few aspects coming out as unique in the current study.

5. Increased risk of accidents due to Mal de Debarquement within first four days after coming ashore (as declared by the authors) requires corroboration and statistical evidence which is not brought out in the current paper.

6. Objective assessment of tension headache and tinnitus has not been done in the study and is totally based on patient description which may lead to erroneous conclusions bringing in subjectivity to the research.

7. Having included women in the study may lead to errors in assessment, and separate criteria for assessment could have been included for women and a descriptive data analysis of impact of being at sea for women needs to be furnished. A comparative data analysis between men and women who have been at sea for the same period of time with constants of type of vessel, age profile and experience at sea may throw more light on the impact of seasickness among Icelandic ‘sea-persons’.

8. Lines 229 to 232 are contradictory in nature. It’s a known fact that tinnitus is more common under age 50 while hearing loss is common in seamen over 50 years of age. However, the authors state the opposite. This needs to be addressed.

9. The study concludes a higher proportion of subjects having migraine compared to a European study, however, the nature of the cited European study is obscure, viz., sample size, type of study, controls etc. Thus, this comparison is fallacious till such time both the current and European studies have been done under similar if not exact circumstances.

10. The study purports in line 160-161 that those who were out at sea for a week or less were more likely to experience seasickness symptoms than those who were out for more than a week (p=0.009) however a contrary statement is made in line 164-167 that both tinnitus and hearing loss appear to increase in accord with length of sea journey, although no significant difference was found (p>0.05). Nor was any significant difference found between length of sea journey and seasickness-related symptoms (p>0.05). This needs deliberation.

11. The data analysis does support the conclusion that seasickness and seasickness like symptoms along with migraine, tinnitus and tension headache are all common features in individuals who venture at sea. However, nothing new has emerged out of this questionnaire-based study except for the fact that further research needs to be done to corroborate various evidence of accidents ashore, types and sizes of vessels, duration of sea, gender differences, age variations among other factors to validate the correlation between trysts at sea with sea sickness.

12. Statistical analysis is basic with use of SPSS ver 25. It would be prudent to bring out correlational study graphs, pie charts and a comprehensive correlational analysis to establish the soundness of the questionnaire. Further, results of Chi Square, Phi and Cramer’s V coefficient may be brought out in detail to solidify the statistical claims. Although the statistical analysis has been performed appropriately, the reviewer needs to be presented with detailed results.

13. Authors have not made all the data underlying the findings available in the manuscript. Questionnaire is not attached and pilot survey if conducted is not described.

Reviewer #2: This paper successfully conducted a questionnaire survey on 262 of the 376 male crew members, and investigated their age, BMI, disease history, seasickness, etc., to understand the working environment and current situation of Icelandic crew members, which has certain research significance, but there are also some shortcomings. :

1. This article is not innovative enough.

2.The reliability of the questionnaire is not high, the subjective factors are mixed, and the individual assessment is inaccurate.

3. 30% of citations are from 10 years ago, which is relatively old.

4. The picture is not clear and the figure lenged is missing.

5. The citation format of literature 32,39 is different from other literatures.

Reviewer #3: The present manuscript describes the results of a survey that was done among Icelandic seamen with regards to their level of seasickness, health-related factors, and their work environment. More than 260 people completed the survey and offered an interesting insight into the effect of seasickness among this group. The rationale of the study is clear and the procedure sound. However, I have some concerns regarding the presentation of the methods and results that need to be addressed by the authors.

Major concerns:

1. Methods: The structure of the questionnaire and the inquired items remain unclear. The authos say that “Questions were asked about working conditions at sea, history of seasickness (seasickness, mal de debarquement and seasickness symptoms), health, demographic background variables…” (p6). This is not enough detail to really understand what was measured in this survey and how it was structured. As this is the main component of the study, the questionnaire needs to be described with mor details. For instance, what is the difference between seasickness and seasickness symptoms? What was the scale to measure seasickness, simply binary “yes/no” or with more details such as “never/sometimes/often”? What do health measures include and why were these items specifically chosen? In addition to describing the main components of the questionnaire, the authors could consider adding the full questionnaire as additional material to the manuscript.

2. Results:

a. The objective of the study was to “…explore the impact of seasickness and mal de debarquement on seamen’s health…” (p5). This makes sense, but the way this objective was tackled in the results section does not really address this objective. The authors chose to run a large number of chi-squared tests to compare different groups, but these tests simply compare different groups (sometimes based on age, sometimes based on experience, sometimes based on position on the vessel etc.). There is no clear rationale offered why these groups were compared (except for age). In order to explore the impact of seasickness on health, it seems more logical to run regression analysis with sickness measures (and other factors such as age, duration on sea etc.) as predictive factors and health-related factors as outcome variables.

b. Only p-values are provided as statistical outcome. This is not sufficient; full statistics (degrees of freedom, effect size) should be included as well in order to better understand how meaningful these group differences are. With a large sample size, even small effects will become significant, but this does not mean that these differences are meaningful.

c. The authors mention that correlations were performed between the groups. I cannot find a summary of these correlations in the manuscript, except for the ones that were explicitly mentioned. I would prefer to see a summary of the correlations, even if they failed significance. Also, why were more common correlation coefficients such as Spearman correlations not used?

d. The survey offers a lot of information. However, the results are not very well structured in the manuscript; instead, the authors list a lot of findings and p-values for a variety of group comparisons (see comment above). I suggest to structure these findings more clearly and present them in subsections that address each factor of interest separately (e.g., seasickness symptoms, health etc.).

e. The figures should be optimized. At least, the SD or Sem should be included. More recently, it has become more common to present the data in a more informative way that indicates the variability and/or shows the individual data points (violin plots, box plots). A bar graph with no further information only provides little information.

Other comments:

1. What was the exact number of male and female seamen in the survey?

2. With regards to terminology, is there a more gender-neutral term instead of seamen (this is not my area of expertise, just wondering). According to a quick google search is seems that some countries (e.g., Canada) have decided to drop the term seamen and now generally refer to them as sailors to be more gender inclusive. Might be worth considering.

3. A large number of tests between two groups were calculated, where the p values adjusted accordingly?

**********

6. PLOS authors have the option to publish the peer review history of their article (what does this mean?). If published, this will include your full peer review and any attached files.

If you choose “no”, your identity will remain anonymous but your review may still be made public.

**Do you want your identity to be public for this peer review?** For information about this choice, including consent withdrawal, please see our Privacy Policy.

Reviewer #1: No

Reviewer #2: No

Reviewer #3: No

[NOTE: If reviewer comments were submitted as an attachment file, they will be attached to this email and accessible via the submission site. Please log into your account, locate the manuscript record, and check for the action link "View Attachments". If this link does not appear, there are no attachment files.]

While revising your submission, please upload your figure files to the Preflight Analysis and Conversion Engine (PACE) digital diagnostic tool, https://pacev2.apexcovantage.com/. PACE helps ensure that figures meet PLOS requirements. To use PACE, you must first register as a user. Registration is free. Then, login and navigate to the UPLOAD tab, where you will find detailed instructions on how to use the tool. If you encounter any issues or have any questions when using PACE, please email PLOS at figures@plos.org. Please note that Supporting Information files do not need this step.

Attachment Submitted filename: PONE_review_renu.docx Click here for additional data file.

# Author comment: Author response to Decision Letter 0

**DOI:** 10.1371/journal.pone.0273477.r002

27 Jul 2022

Thank you for your valuable comments. Responses to all comments are attached in a file (answers to all reviewers), our answers are written in italic.

Attachment Submitted filename: Answers to all reviewers2._july.docx Click here for additional data file.

# Editor report: Decision Letter 1

**DOI:** 10.1371/journal.pone.0273477.r003

**Authors:** Andreas C. Bryhn

1 Aug 2022

PONE-D-21-29503R1Seasickness among Icelandic seamenPLOS ONE

Dear Dr. Arnardottir,

Thank you for submitting your manuscript to PLOS ONE. After careful consideration, we feel that it has merit but does not fully meet PLOS ONE’s publication criteria as it currently stands. Therefore, we invite you to submit a revised version of the manuscript that addresses the points raised during the review process.

==============================

ACADEMIC EDITOR: Dear authors, Thank you for submitting an improved manuscript with great potential. However, according to this journal's publication criteria, all manuscripts must have perfect English before acceptance. I have detected several spelling and grammar errors in the current version. Please have it proof-read by a native English speaker before resubmission.

==============================

Please submit your revised manuscript by September 15. If you will need more time than this to complete your revisions, please reply to this message or contact the journal office at plosone@plos.org. When you're ready to submit your revision, log on to https://www.editorialmanager.com/pone/ and select the 'Submissions Needing Revision' folder to locate your manuscript file.

Please include the following items when submitting your revised manuscript:

- A rebuttal letter that responds to each point raised by the academic editor and reviewer(s). You should upload this letter as a separate file labeled 'Response to Reviewers'.
- A marked-up copy of your manuscript that highlights changes made to the original version. You should upload this as a separate file labeled 'Revised Manuscript with Track Changes'.
- An unmarked version of your revised paper without tracked changes. You should upload this as a separate file labeled 'Manuscript'.

If you would like to make changes to your financial disclosure, please include your updated statement in your cover letter. Guidelines for resubmitting your figure files are available below the reviewer comments at the end of this letter.

If applicable, we recommend that you deposit your laboratory protocols in protocols.io to enhance the reproducibility of your results. Protocols.io assigns your protocol its own identifier (DOI) so that it can be cited independently in the future. For instructions see: https://journals.plos.org/plosone/s/submission-guidelines#loc-laboratory-protocols. Additionally, PLOS ONE offers an option for publishing peer-reviewed Lab Protocol articles, which describe protocols hosted on protocols.io. Read more information on sharing protocols at https://plos.org/protocols?utm_medium=editorial-email&utm_source=authorletters&utm_campaign=protocols.

We look forward to receiving your revised manuscript.

Kind regards,

Andreas C. Bryhn

Academic Editor

PLOS ONE

Journal Requirements:

Please review your reference list to ensure that it is complete and correct. If you have cited papers that have been retracted, please include the rationale for doing so in the manuscript text, or remove these references and replace them with relevant current references. Any changes to the reference list should be mentioned in the rebuttal letter that accompanies your revised manuscript. If you need to cite a retracted article, indicate the article’s retracted status in the References list and also include a citation and full reference for the retraction notice.

[Note: HTML markup is below. Please do not edit.]

[NOTE: If reviewer comments were submitted as an attachment file, they will be attached to this email and accessible via the submission site. Please log into your account, locate the manuscript record, and check for the action link "View Attachments". If this link does not appear, there are no attachment files.]

While revising your submission, please upload your figure files to the Preflight Analysis and Conversion Engine (PACE) digital diagnostic tool, https://pacev2.apexcovantage.com/. PACE helps ensure that figures meet PLOS requirements. To use PACE, you must first register as a user. Registration is free. Then, login and navigate to the UPLOAD tab, where you will find detailed instructions on how to use the tool. If you encounter any issues or have any questions when using PACE, please email PLOS at figures@plos.org. Please note that Supporting Information files do not need this step.

# Author comment: Author response to Decision Letter 1

**DOI:** 10.1371/journal.pone.0273477.r004

9 Aug 2022

Academic Editor, Mr. Andreas C. Bryhn: 

(August 4th): We have had an additional native English speaker reading through the manuscript again. We hope that the manuscript is now in proper English. In our submission today we have updated “Revised Manuscript with Track Changes” which indicates changes made in the manuscripts, “Manuscript” referring to a clean version of the revised manuscript as well as this file “Response to Reviewers”. Other files are the same since our earlier resubmission.

Reviewer #1: 

Thank you for your comments. All changes in the manuscript are highlighted in yellow to make more visible for the reviewers.

1. The research is a simple questionnaire based cross-sectional study whose conclusions are based on responses to a 90-question questionnaire of subjects who have volunteered to participate in the project. Thus, assessment may be considered subjective depending on the state of mind and condition of individuals who have consented to provide data. To bring in objectivity, the study could have included physical examination of subjects as well as covered the assessed conclusions by validating data over a period of time.

Thank you for an excellent suggestion, we have actually planned to do a follow up study on seamen and preventative procedures for seasickness, where physical examination will be conducted.

We have included more details about the participants, see line 106-107 in yellow.

2. The questionnaire used has not been published along with the manuscript for the reviewer to objectively assess the interpretation of data. Also, the questionnaire has been validated not by a pilot study but by the mere improvement of an already existing questionnaire which was used in earlier research. Thus, objective validation of the questionnaire needs to be done along with a prior pilot assessment of a fixed number of subjects before undertaking the actual research. The technical soundness of the research may not be possible to comment upon since the questionnaire is not available at the present period.

More detailed description on the questionnaire has been added in lines 114-117 in yellow. An English version of the questionnaire (questions used) has been added as an appendix (referred to in line 116-117). Further questions were based on MSSQ short.

3. Although cross-sectional in nature, lack of controls in the study renders the comparative analysis of data impossible.

We agree that that is the golden standard, but the purpose of this study was to explore the impact of seasickness and mal de débarquement (MdD) on seamen´s health, rather that comparing them with other professions. We also questioned how valid comparison between two different professions would be. 

4. Conclusions drawn based on the data collated reflects earlier studies in the same arena, with only a few aspects coming out as unique in the current study.

Even though sea sickness and MdD has been reported high in Icelandic seamen before, as far as we know, 1) symptoms of seasickness is current in majority of seamen that still deny that they are seasick, but clearly suffer from seasickness symptoms, 2) It is correct that migraine and tension headache are common symptoms in individuals who venture at sea, but in the same way important in sailors working at sea. Seamen, that work at sea are unfortunately not immune to those symptoms that occur in their working environment. We found increase proportion of headache in seamen compared to general cohort which gives us important information to look closer into e.g. in relation to their working environment. 3) no study has before addressed the increased admission to hospital ashore within seamen (see line 227-232). We believe that these facts increase the novelty of this paper.

5. Increased risk of accidents due to Mal de débarquement within first four days after coming ashore (as declared by the authors) requires corroboration and statistical evidence which is not brought out in the current paper.

Here we have come across a misunderstanding from the reviewer which was really important to have the opportunity to clarify. Our results did not mention any certain days. We only mention these four days as a suggestion for future studies concerning accidents and MdD. This is now part of “Regarding seamen…” lines (287). Time frame of 4 days is used as this is the time needed to gain (adapt) sea legs when going at sea and loosing them when coming ashore (adapt to fixed terrestrial environment) see Stoffregen, T. A., et al. (2013). "Getting Your Sea Legs." PLoS One 8(6): e66949.

6. Objective assessment of tension headache and tinnitus has not been done in the study and is totally based on patient description which may lead to erroneous conclusions bringing in subjectivity to the research.

Thank you for your comment, we totally agree. We have now added this to the discussion of the study weakness, to the section we addressed their subjective analyze of their health condition (see line 278-286). We have also added some extra clarifications in lines 147 to 149 to make this clearer. 

7. Having included women in the study may lead to errors in assessment, and separate criteria for assessment could have been included for women and a descriptive data analysis of impact of being at sea for women needs to be furnished. A comparative data analysis between men and women who have been at sea for the same period of time with constants of type of vessel, age profile and experience at sea may throw more light on the impact of seasickness among Icelandic ‘sea-persons’.

Thank you for your suggestion. We wanted to look at seamen as a group, women included (sea-persons � great thank you), even though women are a minority group of seamen. We ran the analysis excluding women, but that did not result in any significant difference. We also added some extra details about gender ratio in lines 131-132.

8. Lines 229 to 232 are contradictory in nature. It’s a known fact that tinnitus is more common under age 50 while hearing loss is common in seamen over 50 years of age. However, the authors state the opposite. This needs to be addressed.

We believe that there has been a misunderstanding. We say (see line 248-249 in the revised manuscript): “Seamen aged under 50 were more likely to experience tinnitus, while those over 50 were more likely to report hearing loss” This also point to the known fact that tinnitus is often the first symptom of noise related hearing loss.

9. The study concludes a higher proportion of subjects having migraine compared to a European study, however, the nature of the cited European study is obscure, viz., sample size, type of study, controls etc. Thus, this comparison is fallacious till such time both the current and European studies have been done under similar if not exact circumstances.

We are aware of this weakness and address this in the weakness chapter (lines 286-296) as well as changing wording in lines regarding those two studies mentioned. 

10. The study purports in line 160-161 that those who were out at sea for a week or less were more likely to experience seasickness symptoms than those who were out for more than a week (p=0.009) however a contrary statement is made in line 164-167 that both tinnitus and hearing loss appear to increase in accord with length of sea journey, although no significant difference was found (p>0.05). Nor was any significant difference found between length of sea journey and seasickness-related symptoms (p>0.05). This needs deliberation.

Thank you for the comment. This has now been clarified, see e.g. lines 173-175.

11. The data analysis does support the conclusion that seasickness and seasickness like symptoms along with migraine, tinnitus and tension headache are all common features in individuals who venture at sea. However, nothing new has emerged out of this questionnaire-based study except for the fact that further research needs to be done to corroborate various evidence of accidents ashore, types and sizes of vessels, duration of sea, gender differences, age variations among other factors to validate the correlation between trysts at sea with sea sickness.

Thank you for your comment. It is correct that migraine and tension headache are common symptoms in individuals who venture at sea, but in the same way important in sailors working at sea. Seamen, that work at sea are unfortunately not immune to those symptoms that occur in their working environment. We found increase proportion of headache in seamen compared to general cohort which gives us important information to look closer into e.g. in relation to their working environment. Tinnitus is an injury on the cochlea and predecessor of hearing loss and is important to study further in relation to seamen´s working environment. Former studies that have studied seasickness have usually used subjective methods and asked “have you ever felt seasickness”, but when sailors are pressed further and asked about symptoms that classifies as symptoms of seasickness at sea, more sailors are in fact seasick. This is a new approach in determine seasickness. We have reworded the end of the paper and conclusion to address this better.

12. Statistical analysis is basic with use of SPSS ver 25. It would be prudent to bring out correlational study graphs, pie charts and a comprehensive correlational analysis to establish the soundness of the questionnaire. Further, results of Chi Square, Phi and Cramer’s V coefficient may be brought out in detail to solidify the statistical claims. Although the statistical analysis has been performed appropriately, the reviewer needs to be presented with detailed results.

Thank you for this suggestion. We have improved our result chapter in accordance to your comment and added appropriated coefficients. Se changes in the result chapter in yellow.

13. Authors have not made all the data underlying the findings available in the manuscript. Questionnaire is not attached and pilot survey if conducted is not described.

We have provided an English version of the questionnaire (see Appendix 1), to clarify how data was collected and which questions were asked. Data has as well been made available to repeat analysis if wished (see attached SPSS file). 

Reviewer #2: 

This paper successfully conducted a questionnaire survey on 262 of the 376 male crew members, and investigated their age, BMI, disease history, seasickness, etc., to understand the working environment and current situation of Icelandic crew members, which has certain research significance, but there are also some shortcomings. :

Thank you for your comments. All changes in the manuscript are highlighted in yellow to make more visible for the reviewers. 

1. This article is not innovative enough: 

Thank you for your comment. Even though sea sickness and MdD has been reported high in Icelandic seamen before, as far as we know, 1) symptoms of seasickness is current in majority of seamen that still deny that they are seasick, but clearly suffer from seasickness symptoms, 2) It is correct that migraine and tension headache are common symptoms in individuals who venture at sea, but in the same way important in sailors working at sea. Seamen, that work at sea are unfortunately not immune to those symptoms that occur in their working environment. We found increase proportion of headache in seamen compared to general cohort which gives us important information to look closer into e.g. in relation to their working environment. 3) no study has before addressed the increased admission to hospital ashore within seamen (see line 228-231). We believe that these facts increase the novelty of this paper.

2. The reliability of the questionnaire is not high, the subjective factors are mixed, and the individual assessment is inaccurate. 

Thank you for your comment. Motion sickness and it´s important part seasickness is and has been validated through questionnaires in all studies. Our questionnaire is well known and used in Iceland and has been tested earlier and in accordance to other studies on seasickness. We find the subjective factors clear and fixed and individual assessment accurate.

3. 30% of citations are from 10 years ago, which is relatively old. 

Seasickness has not been examined sufficiently in recent years, which gives the reason for the age of the references. We did though add one reference (ref 33). The novelty of this study is that we have subjects that have worked at sea for several years and such results has been missing in studies on seasickness. We have made this clearer in lines 278-279.

4. The picture is not clear and the figure lenged is missing. 

Thank you for your suggestion. Figure legends have been expanded and the figures made clearer.

5. The citation format of literature 32,39 is different from other lituratures. 

These are Icelandic web pages. We have now put English titles with the Icelandic one´s in the reference list (see references 32 and 40 in yellow).

Reviewer #3: The present manuscript describes the results of a survey that was done among Icelandic seamen with regards to their level of seasickness, health-related factors, and their work environment. More than 260 people completed the survey and offered an interesting insight into the effect of seasickness among this group. The rationale of the study is clear and the procedure sound. However, I have some concerns regarding the presentation of the methods and results that need to be addressed by the authors.

Major concerns:

Thank you for your comments. All changes in the manuscript are highlighted in yellow to make more visible for the reviewers.

1. Methods: The structure of the questionnaire and the inquired items remain unclear. The authos say that “Questions were asked about working conditions at sea, history of seasickness (seasickness, mal de debarquement and seasickness symptoms), health, demographic background variables…” (p6). This is not enough detail to really understand what was measured in this survey and how it was structured. As this is the main component of the study, the questionnaire needs to be described with mor details. For instance, what is the difference between seasickness and seasickness symptoms? What was the scale to measure seasickness, simply binary “yes/no” or with more details such as “never/sometimes/often”? What do health measures include and why were these items specifically chosen? In addition to describing the main components of the questionnaire, the authors could consider adding the full questionnaire as additional material to the manuscript.

Thank you for the suggestion. More detailed description on the questionnaire has been added in lines 114-117 in yellow. An English version of the questionnaire (questions used) has been added as an appendix (referred to in line 116-117). Further questions were based on MSSQ short.

2. Results:

a. The objective of the study was to “…explore the impact of seasickness and mal de debarquement on seamen’s health…” (p5). This makes sense, but the way this objective was tackled in the results section does not really address this objective. The authors chose to run a large number of chi-squared tests to compare different groups, but these tests simply compare different groups (sometimes based on age, sometimes based on experience, sometimes based on position on the vessel etc.). There is no clear rationale offered why these groups were compared (except for age). In order to explore the impact of seasickness on health, it seems more logical to run regression analysis with sickness measures (and other factors such as age, duration on sea etc.) as predictive factors and health-related factors as outcome variables.

Thank you for a good and valid comment. Because of the nature of the questionnaire, nominal variables were most common type variables obtained. After consulting a statistician, it was decided that it would be best to describe the data with Chi-square test (in most cases), but after further consulting we have improved our result chapter in accordance to your comment and added appropriated coefficients. Se changes in the result chapter in yellow.

We can´t say that seasickness is causing this or that, but we can draw the conclusion that the frequency of headache is high. The questionnaire suggests that health problems such as headache (tension and migraine), tinnitus and seasickness symptoms are clearly factors that are closely related to the working environment at sea. The duration of how long the seaman been at sea gives us a reason to interpret that this hostile working environment is contributing to the incidence of these health problems.

b. Only p-values are provided as statistical outcome. This is not sufficient; full statistics (degrees of freedom, effect size) should be included as well in order to better understand how meaningful these group differences are. With a large sample size, even small effects will become significant, but this does not mean that these differences are meaningful.

Thank you for an excellent suggestion. We have improved our result chapter in accordance to your comment and added appropriated coefficients. Se changes in the result chapter in yellow.

c. The authors mention that correlations were performed between the groups. I cannot find a summary of these correlations in the manuscript, except for the ones that were explicitly mentioned. I would prefer to see a summary of the correlations, even if they failed significance. Also, why were more common correlation coefficients such as Spearman correlations not used?

Thank you for your comment. We have added phi coefficient in line 168 to make the result clearer. We chose Phi and Cramer´s as we had nominal data (yes/no answers in both variables). 

d. The survey offers a lot of information. However, the results are not very well structured in the manuscript; instead, the authors list a lot of findings and p-values for a variety of group comparisons (see comment above). I suggest to structure these findings more clearly and present them in subsections that address each factor of interest separately (e.g., seasickness symptoms, health etc.).

The result chapter has been reorganized according to your suggestions, see subtitles in result chapter in yellow.

e. The figures should be optimized. At least, the SD or Sem should be included. More recently, it has become more common to present the data in a more informative way that indicates the variability and/or shows the individual data points (violin plots, box plots). A bar graph with no further information only provides little information.

We have improved the presentation of the figures. Figure 2 has been put in a table as a suggestion for reviewers (could be an appendix if needed). Because of the nature of the data, SD can´t be calculated sufficiently (percentage of yes/no answers – SD would not be descriptive, but would apply if we had e.g. mean/average). 

Other comments:

1. What was the exact number of male and female seamen in the survey? 

Information added in results, line 132-133.

2. With regards to terminology, is there a more gender-neutral term instead of seamen (this is not my area of expertise, just wondering). According to a quick google search is seems that some countries (e.g., Canada) have decided to drop the term seamen and now generally refer to them as sailors to be more gender inclusive. Might be worth considering. 

Thank you for your interesting comment. The term “madur” (sjomadur = seamen) in Icelandic is not gender specific, has to do with both men and women. We considered “sailor” but refers more to those who sail as a part of a hobby. Our future studies aim to study women only – and maybe we will find a good term for them in that study.

3. A large number of tests between two groups were calculated, where the p values adjusted accordingly? 

We have improved the presentation of the result chapter and hope that it will answer your question.

Attachment Submitted filename: Responce to Reviewers.docx Click here for additional data file.

# Editor report: Decision Letter 2

**DOI:** 10.1371/journal.pone.0273477.r005

**Authors:** Andreas C. Bryhn

10 Aug 2022

Seasickness among Icelandic seamen

PONE-D-21-29503R2

Dear Dr. Arnardottir,

We’re pleased to inform you that your manuscript has been judged scientifically suitable for publication and will be formally accepted for publication once it meets all outstanding technical requirements.

Within one week, you’ll receive an e-mail detailing the required amendments. When these have been addressed, you’ll receive a formal acceptance letter and your manuscript will be scheduled for publication.

An invoice for payment will follow shortly after the formal acceptance. To ensure an efficient process, please log into Editorial Manager at http://www.editorialmanager.com/pone/, click the 'Update My Information' link at the top of the page, and double check that your user information is up-to-date. If you have any billing related questions, please contact our Author Billing department directly at authorbilling@plos.org.

If your institution or institutions have a press office, please notify them about your upcoming paper to help maximize its impact. If they’ll be preparing press materials, please inform our press team as soon as possible -- no later than 48 hours after receiving the formal acceptance. Your manuscript will remain under strict press embargo until 2 pm Eastern Time on the date of publication. For more information, please contact onepress@plos.org.

Kind regards,

Andreas C. Bryhn

Academic Editor

PLOS ONE

Additional Editor Comments (optional):

Reviewers' comments:

# Editor report: Acceptance letter

**DOI:** 10.1371/journal.pone.0273477.r006

**Authors:** Andreas C. Bryhn

15 Aug 2022

PONE-D-21-29503R2 

Seasickness among Icelandic seamen 

Dear Dr. Arnardottir:

I'm pleased to inform you that your manuscript has been deemed suitable for publication in PLOS ONE. Congratulations! Your manuscript is now with our production department. 

If your institution or institutions have a press office, please let them know about your upcoming paper now to help maximize its impact. If they'll be preparing press materials, please inform our press team within the next 48 hours. Your manuscript will remain under strict press embargo until 2 pm Eastern Time on the date of publication. For more information please contact onepress@plos.org.

If we can help with anything else, please email us at plosone@plos.org. 

Thank you for submitting your work to PLOS ONE and supporting open access. 

Kind regards, 

PLOS ONE Editorial Office Staff

on behalf of

Dr. Andreas C. Bryhn 

Academic Editor

PLOS ONE
